# Exploring Polymeric Micelles for Improved Delivery of Anticancer Agents: Recent Developments in Preclinical Studies

**DOI:** 10.3390/pharmaceutics5010201

**Published:** 2013-03-22

**Authors:** Chalet Tan, Yingzhe Wang, Wei Fan

**Affiliations:** Cancer Nanomedicine Laboratory, Department of Pharmaceutical Sciences, Mercer University, Atlanta, GA 30341, USA; E-Mails: Yingzhe.Wang@live.mercer.edu (Y.W.); stojkovic@163.com (W.F.)

**Keywords:** block polymer, EPR effect, tumor-targeting ligand, pharmacokinetics, targeted cancer therapy

## Abstract

As versatile drug delivery systems, polymeric micelles have demonstrated particular strength in solubilizing hydrophobic anticancer drugs while eliminating the use of toxic organic solvents and surfactants. However, the true promise of polymeric micelles as drug carriers for cancer therapy resides in their potential ability to preferentially elevate drug exposure in the tumor and achieve enhanced anticancer efficacy, which still remains to be fully exploited. Here, we review various micellar constructs that exhibit the enhanced permeation and retention effect in the tumor, the targeting ligands that potentiate the anticancer efficacy of micellar drugs, and the polyplex micelle systems suitable for the delivery of plasmid DNA and small interference RNA. Together, these preclinical studies in animal models help us further explore polymeric micelles as emerging drug carriers for targeted cancer therapy.

## 1. Introduction

Self-assembled from biodegradable and biocompatible amphiphilic block polymers and with sizes ranging 10–200 nm, polymeric micelles are promising nanocarriers for anticancer drugs, as attested by a number of such formulations currently in clinical trials [1]. There are several reasons why polymeric micelles are well-suited for cancer therapy. First, a number of potent anticancer drugs are water-insoluble and require the use of toxic organic solvents/surfactants for solubilization, which severely exacerbate the dose-limiting toxicities of the therapies. Owing to their unique core-shell structure, polymeric micelles have the capacity to solubilize clinically relevant doses of the hydrophobic drugs without the inclusion of any organic solvent, thereby greatly improving the safety of these drugs. Second, polymeric micelles can preferentially accumulate in the tumor via the enhanced permeability and retention (EPR) effect [2]. Due to the leaky vasculature and dysfunctional lymphatic drainage in the tumor tissues, nano-sized drug carriers preferentially extravasate across the tumor endothelium and elevate the drug exposure in the tumor, while having very limited access to normal organs because of their tightly-knitted endothelial lining. Finally, polymeric micelles are amenable to the surface modification with the targeting ligands, which specifically recognize the receptors overexpressed on the surface of tumor cells and/or tumor endothelium, resulting in highly efficient intracellular delivery of micellar drugs [3].

**Figure 1:**
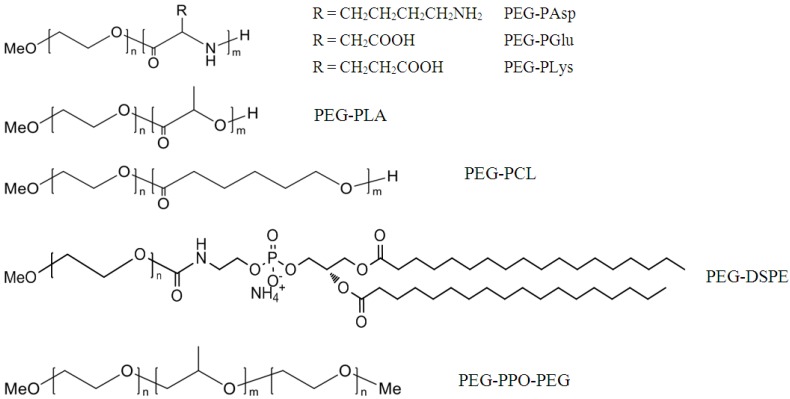
Chemical structures of the commonly studied block polymers.

A micelle consists of a corona formed by the hydrophilic blocks extending into the aqueous solution and a core formed by the hydrophobic segments. The hydrophilic block is almost exclusively poly(ethylene glycol) (PEG), because of its excellent biocompatibility and “stealth” property that minimize undesirable interactions with serum proteins and cellular components. On the other hand, the composition of the hydrophobic block varies enormously, which can be tailored to encapsulate drug molecules with a wide variety of structures, lipophilicity and charges, contributing to the versatility of polymeric micelles as drug carriers. The frequently studied block polymers include PEG-poly(amino acids), PEG-poly(d,l-lactide) (PEG-PLA), PEG-poly(ε-caprolactone) (PEG-PCL), PEG-distearoylphosphatidylethanolamine (PEG-DSPE) and PEG-poly(propyl oxide)-PEG (PEG-PPO-PEG, Pluronics^®^), as listed in Figure 1.

There are excellent reviews on general principles and applications of polymeric micelles as drug carriers [4,5,6]. Here, we aim to present a concise overview of recent preclinical studies on block polymeric micelles for the delivery of anticancer agents. In particular, we focus on the micellar carriers that physically entrap drugs within the micellar core, which have improved the pharmacokinetics and anticancer efficacy of the drugs in animal models.

## 2. Polymeric Micelles Can Improve Anticancer Efficacy of Hydrophobic Drugs via Passive Targeting

Polymeric micelles are capable of delivering anticancer agents passively to the tumor sites via the EPR effect with two key prerequisites. First, polymeric micelles themselves need to maintain their structurally integrity in the circulation for extended period of time. Second, the drug molecules need to be retained within the micelles to eventually extravasate with the carriers into the tumor space. A number of micellar constructs have been shown to enhance the efficacy of hydrophobic drugs, as summarized in Table 1. 

**Table 1:** Drug-loaded polymeric micelles can enhance the anticancer efficacy of the drugs.

**Polymer**	**Drug**	**Size (nm)**	**Tumor Model**	**Effects** *(compared to the free drug)*	**Ref.**
PEG-PLA	β-lapachone	30	s.c. A549	↑ drug levels in plasma and the tumor ↓ tumor growth ↑ survival time	[7]
			orthotopic LLC	↓ metastatic tumor burden ↑ survival time	
PEG-PLA & P85	docetaxel	21	s.c. KB_v_	↑ drug levels in plasma and the tumor ↓ tumor growth	[8]
PEG-DSPE	doxorubicin	10–20	s.c. & orthotopic LLC	↑ drug levels in plasma and the tumor	[9]
			s.c. 4T1	↓ tumor growth ↑ survival time	
PEG-DSPE	docetaxel	21	s.c. MCF-7	↑ drug levels in plasma and the tumor ↓ tumor growth	[10]
PEG-DSPE	paclitaxel & 17-AAG	11	s.c. SKOV-3	↑ drug levels in plasma and the tumor ↓ tumor growth	[11]
PEG-poly(benzyl aspartate)	camptothecin	192	s.c. colon 26	↓ tumor growth	[12]
			s.c. B16BL6	↑ drug levels in plasma	
	fenretinide	173		↓ tumor growth	[13]
PEG-PCL-PEG	paclitaxel	93	s.c. EMT6	↑ drug levels in plasma ↓ tumor growth	[14]
chlorin-conjugated PCL-PEG	SN-38	130	s.c. HT-29	↑ drug levels in plasma and the tumor ↓ tumor growth ↑ survival time	[15]
P123/F127	paclitaxel	24		↑ drug levels in plasma and the tumor ↑ survival time	[16]
P105/PCL_50_	paclitaxel	149	s.c. SKOV-3	↓ tumor growth	[17]
CP750	*cis*-(cha)_2_-Pt(NO3)_2_	7	s.c. B16F10	↑ drug levels in plasma and the tumor without tumor growth inhibition	[18]
	docetaxel		s.c. MDA-MB-231 & s.c. MNK-28	no improvement in pharmacokinetics ↓ tumor growth	[19]
PEG-PAE	doxorubicin	62	s.c.B16F10	↓ tumor growth	[20,21]
	camptothecin	214	s.c. MDA-MB-231	↑ survival time	
PEG^5K^-CA_8_	paclitaxel	20–60	s.c. & i.p.SKOV-3	↑ drug levels in plasma and the tumor ↓ tumor growth ↑ survival time	[22]
PEG^2K^-CA_4_	doxorubicin	12	s.c. Raji lymphoma	↓ tumor growth ↑ survival time	[23]

### 2.1. PEG-PLA-Based Micelles

In a study by Blanco *et al.* PEG-PLA micelles were utilized to formulate β-lapachone (β-lap), a poorly water-soluble anticancer agent [7]. Although the pharmacokinetics of β-lap was not studied directly, the authors found that ^3^H-labled β-lap-loaded PEG-PLA micelles had long circulation time and accumulated substantially in the tumor. Owing to its stable incorporation with the PLA micellar core, β-lap was likely retained within the micelles in the blood stream for a prolonged period of time and therefore could achieve enhanced drug accumulation in the tumor via the EPR effect. In both subcutaneous non-small cell lung cancer (NSCLC) A549 and orthotopic Lewis lung carcinoma (LCC) models, β-lap micelles exhibited superior anti-cancer efficacy in terms of suppressing tumor growth and extending survival time, compared to a formulation undergoing clinical testing which complexed β-lap with cyclodextran. 

Mu *et al.* reported that in mice bearing multidrug resistant (MDR) oral epidermal carcinoma KB_v_ s.c. xenografts, docetaxel-loaded PEG-PLA (MPP) micelles prolonged the circulation time of docetaxel and reduced tumor growth more effectively than Taxotere^®^, the clinically approved formulation of docetaxel [8]. Interestingly, the mixed micelles of MPP and Pluronic 85 (P85) at 6:1 (*w*/*w*) ratio could deliver docetaxel and retard the tumor growth more effectively without significantly affecting drug levels in plasma, compared to docetaxel carried by MPP micelles. The authors attributed the augmented efficacy of docetaxel-loaded MPP/P85 micelles to the inhibitory activity against P-gp by P85. 

### 2.2. PEG-DSPE-Based Micelles

Liang and his coworkers investigated the delivery of doxorubicin in PEG-DSPE micelles [9]. Doxorubicin could be stably incorporated into PEG-DSPE micelles owing to the electrostatic and hydrophobic interactions. Results from the pharmacokinetic analysis in mice bearing LLC s.c. xenografts indicated that micellar doxorubicin caused about 10-fold and 5-fold increase in AUC of drug concentrations in plasma and tumors, respectively, when compared to free doxorubicin. Importantly, in both subcutaneous and pulmonary LLC models, doxorubicin-loaded PEG-DSPE micelles were significantly more effective than the free drug in suppressing the tumor growth. 

In a study by Tong *et al.* PEG-DSPE micelles were employed to deliver docetaxel [10]. It was shown that docetaxel-loaded micelles increased the drug exposure in plasma by about 50% compared to Taxotere^®^. In mice bearing breast cancer MCF-7 s.c. xenografts, docetaxel micelles retarded the tumor growth to a greater extent than Taxotere^®^.

Katragadda *et al.* explored the combined delivery of paclitaxel and tanespimycin (17-AAG) in PEG-DSPE micelles [11]. To improve the drug retention within the micelles, TPGS was incorporated to form the mixed micelles. Compared with free paclitaxel and 17-AAG, the administration of the dual drug-loaded PEG-DSPE/TPGS mixed micelles in mice bearing ovarian SKOV-3 s.c. xenografts led to 3.5- and 1.7-fold increase in the tumor concentrations of paclitaxel and 17-AAG, respectively, without significantly affecting drug levels in healthy organs. Enhanced accumulation of the micellar drugs in the tumor was further corroborated by the near infrared (NIR) imaging. Consequently, paclitaxel/17-AAG-loaded micelles caused near-complete arrest of the tumor growth, whereas the antitumor efficacy of the free drug combination was modest and transient. Furthermore, comparative metabolomics profiling of the tumor tissues by proton nuclear magnetic resonance revealed metabolomic alterations that are well correlated with the potent antiproliferative activity of the micellar drugs. 

### 2.3. PEG-Poly(benzyl aspartate)-Based Micelles

Kawano *et al.* demonstrated that PEG-poly(benzyl aspartate) micelles could be used to deliver camptothecin (CPT), a potent yet highly unstable anticancer agent [12]. The results clearly indicated that in mice bearing colon cancer 26 s.c. xenografts, CPT-loaded micelles increased drug levels in plasma and tumor tissues by about 150-fold and 5-fold, respectively, compared to those of free CPT. As a result, CPT-loaded micelles exerted drastically more potent suppression in tumor growth. PEG-poly(benzyl aspartate) micelles were later evaluated as a carrier for fenretinide, a synthetic retinoid with poor aqueous solubility [12]. Marked improvement in the pharmacokinetics and tumor growth retardation was observed with fenretinide-loaded micelles in murine melanoma B16BL6 model when compared to the drug in O/W emulsions. 

### 2.4. PEG-PCL-Based Micelles

Zhang *et al.* evaluated PEG-PCL-PEG (PCEC) micelles as a drug carrier for paclitaxel [14]. It was shown that paclitaxel-loaded PCEC micelles yielded higher drug levels in plasma than Taxol^®^, the clinically approved formulation for paclitaxel that contains large volume of Cremophor EL and ethanol. In mice bearing breast cancer EMT6 s.c. xenografts, paclitaxel-loaded micelles displayed more potent tumor growth inhibition than Taxol^®^. 

Peng *et al.* demonstrated that chlorin-core star-shaped block micelles (CSBC) composed of chlorin-conjugated PEG-PCL, could serve as drug carriers for SN-38, an active and poorly soluble metabolite of irinotecan (CPT-11) [15]. SN-38-loaded CSBC micelles showed about 20 times higher AUC of SN-38 in both plasma and tumor tissues, compared to those of CPT-11. Consequently, SN-38-loaded CSBC micelles exhibited significantly more potent tumor growth suppression than free CPT-11 in a colon cancer HT-29 s.c. xenograft model. Taking advantage of chlorin being a photosensitizer, the anticancer efficacy of SN-38-loaded CSBC was further enhanced when combining the drug therapy with photodynamic therapy. 

### 2.5. PEG-PPO-PEG-Based Micelles

Fang *et al.* developed paclitaxel-loaded mixed micelles consisting of Pluronic P123 and F127 copolymers [16]. In murine melanoma B16F10 s.c. xenograft model, P123/F127 mixed micelles resulted in 2-fold higher paclitaxel exposure in plasma and tumor tissues compared to Taxol^®^, which led to prolonged survival time of the tumor-bearing mice. The efficacy of paclitaxel-loaded mixed micelles was further evaluated in pulmonary B16F10 metastatic mouse model. It was found that the micelle formulation significantly reduced metastases and improved the survival of the tumor-bearing mice. In another study by Fang *et al.* PCL-modified Pluronic P105 (P105/PCL_50_) micelles were examined as drug carriers for paclitaxel [17]. They demonstrated in mice bearing paclitaxel-resistant SKOV-3 s.c. xenografts that, paclitaxel-loaded P105/PCL_50_ micelles were more effective than Taxol^®^ in suppressing the tumor growth, which could at least be partially attributed to the inhibitory activity of Pluronic polymers against P-gp efflux transporter. 

### 2.6. Novel Micelle Constructs

Sohn *et al.* reported a tripodal cyclotriphosphate amphiphile [NP(PEG750)(GlyPheLeu)_2_Et]_3_ (CP750), which self-assembled into stable micelles with the oligopeptides formed the hydrophobic core [18]. By solubilizing *cis-*(cha)_2_-Pt(NO3)_2_, a water-insoluble platinum (II) agent, in the micellar core, the authors demonstrated in mice bearing melanoma B16F10 s.c. xenografts that the drug-loaded CP750 micelles markedly increased the terminal half-life and the systemic exposure of the drug, leading to enhanced drug accumulation in the tumor. In the meantime, the acute toxicity of platinum was greatly reduced. Nevertheless, it was found later that the micelle-encapsulated platinum (II) drug did not inhibit tumor growth *in vivo*, likely due to insufficient drug release in the tumor. Recently, they investigated the delivery of docetaxel using CP570 micelles [19]. It was found that CP570 micelles did not significantly improve the pharmacokinetics of docetaxel. In mice bearing breast cancer MDA-MB-231 and gastric cancer MKN-28 s.c. xenografts, docetaxel-loaded CP570 (5 mg/kg) exhibited equivalent antitumor effect as observed with Taxotere^®^ (15 mg/kg). However, because the efficacy of Taxotere^®^ at 5 mg/kg was not evaluated in the study, it remained inconclusive whether docetaxel-loaded CP570 micelles did indeed improve the efficacy of docetaxel *in vivo*.

Lee and his group synthesized PEG-*b*-poly(β-amino ester) (MPEG-PAE), which formed micelles responsive to weakly acidic tumor environment (pH 6.4) owing to the tertiary amine (pK_b_ 6.5) in PAE [20]. In mice bearing B16F10 s.c. xenografts, doxorubicin-loaded MPEG-PAE micelles caused more severe tumor growth inhibition and prolonged the survival of mice, in comparison with free doxorubicin. Similarly, camptothecin-loaded MPEG-PAE micelles also demonstrated superior antitumor efficacy in MDA-MB-231 tumor-bearing mice [21].

Lam and his coworker developed a series of PEG-oligocholic acid based telodendrimers, which self assembled to form stable micelles and efficiently solubilize hydrophobic drugs. In particular, they reported that PEG^5K^-CA_8_ micelles could solubilize paclitaxel with high loading capacity and stability [22]. Notably, paclitaxel-PEG^5K^-CA_8_ micelles exerted more potent anticancer effect with reduced toxicity compared to Taxol^®^ and Abraxane^®^ in mice bearing subcutaneous and intraperitoneal SKOV-3 tumors, which was attributed to their prolonged circulation time, preferential tumor accumulation and deeper penetration into the tumor tissues, as confirmed by the whole-body NIR imaging. Subsequently, PEG^5K^-CA_8_ and PEG^2K^-CA_4_ micelles were evaluated as drug carriers for doxorubicin [23]. It was found that PEG^2K^-CA_4_ micelles served as better carriers for doxorubicin in terms of the drug retention in the blood stream and drug accumulation in the tumor. As a result, doxorubicin-PEG^2K^-CA_4_ micelles achieved more potent tumor growth inhibition and prolonged survival in subcutaneous Raji lymphoma-bearing mice, compared to free doxorubicin. These studies underscore the importance of compatibility between the micelle core and drugs, which significantly influences the pharmacokinetics and efficacy of the micellar formulations.

## 3. Targeting Ligands Enhance the Anticancer Efficacy of Micellar Drugs

Even though the extravasation and tumor distribution of micellar drugs is generally considered to be dependent on the size and surface charge of the nanocarriers, the presence of tumor cell-targeting ligands on polymeric micelles can mediate the binding and subsequent drug internalization into the tumor cells, resulting in elevated drug accumulation in the tumor and enhanced anticancer efficacy. Various tumor cell-targeting ligands have been conjugated onto the drug-loaded polymeric micelles to facilitate the drug uptake into the tumor cells, as summarized in Table 2. 

**Table 2:** Targeting ligands enhance the anticancer efficacy of micellar drugs.

**Ligand**	**Polymer**	**Drug**	**Size (nm)**	**Tumor Model**	**Effects** *(compared to the nontargeted micelles)*	**Ref.**
folate	PEG-PLLA &PEG-PHis	doxorubicin	94	s.c. 4T1	↓ tumor growth ↓ metastatic tumor burden ↑ survival time	[24]
folate	PEG-poly (His-*co*-Phe) & PEG-PLA	doxorubicin	150	s.c. A2780/Dox^R^	↑ drug levels in the tumor ↓ tumor growth	[25]
folate	Poly(2-HEMA-*co*-His)-*g*-PLA & PEG-PLA	doxorubicin	~200	s.c. HeLa	↓ drug levels in plasma (slightly) ↓ tumor growth	[26]
folate	P123 & F127	paclitaxel	20–50	s.c. KB_v_	↓ drug levels in plasma (slightly) ↓ tumor growth	[27]
folate	PEO_z_-PLA	m-THPC	~100	s.c. KB	↑ drug levels in the tumor ↓ tumor growth	[28]
tarnsferrin	PCL-PEEP	paclitaxel	88	intracranial U87MG	↑ drug levels in the brain ↑ survival time	[29]
cRGD	PEG-PLA	paclitaxel	26	s.c. & intracranial U87MG	↓ tumor growth ↑ survival time	[30]
NGR	PEG-DSPE	paclitaxel	54	intracranial C6	↓ tumor growth	[31]
D-SP5	PEG-DSPE	doxorubicin	30	s.c. KB	↓ tumor growth	[32]
OA02	PEG^5K^-CA_8_	paclitaxel	21	s.c. SKOV-3	↓ tumor growth ↑ survival time	[33]
TAT	PEG-DSPE	paclitaxel	8–25	s.c. 4T1	↑ apoptosis in the tumor	[34]
lanreotide	PEG-PCL	paclitaxel	43	s.c. H446	↑ drug levels in the tumor	[35]
				s.c. MCF-7	↓ tumor growth ↑ survival time	
LyP-1	PEG-PCL	artemisinin	30	orthotopic MDA-MB-435	home to lymphatic vessels ↓ tumor growth	[36]
AP peptide	MPEG-PAE/PEG-PLA	doxorubicin	181	s.c. MDA-MB-231	more rapid accumulation in the tumor and longer retention ↓ tumor growth	[37]

### 3.1. Folic Acid

One of the most investigated tumor-targeting ligands is folic acid, which targets the folate receptor α (FR-α) that is overexpressed in cancers of ovary, breast, lung, kidney, head and neck, and brain [38]. Bae and his coworkers developed pH-sensitive mixed micelles composed of folate-linked PEG-*b*-poly(l-lactide) (folate-PEG-PLLA) and PEG-*b*-poly(l-histidine) (PEG-polyHis) at a weight ratio of 3:7 [24]. The protonation of polyHis block at acidic pH endowed pH sensitivity to these micelles. It was shown in 4T1 s.c. xenograft-bearing mice that doxorubicin carried by PEG-polyHis/folate-PEG-PLLA micelles possessed the most potent anticancer efficacy in terms of tumor growth inhibition, improved survival and reduced metastasis, compared to the free drug, the drug-loaded PEG-polyHis micelles and PEG-PLLA micelles. Additionally, the authors evaluated a second generation of pH-sensitive micelles (PHSM) composed of PEG-*b*-poly(l-histidine-*co*-l-phenylalanine) (PEG-poly(His-*co*-Phe)) and folate-PEG-PLLA for the delivery of doxorubicin [25]. They found that even though doxorubicin carried by PHSM and PH-insensitive folate-PEG-PLLA micelles (PHIM) both showed 3-fold higher plasma drug concentrations than free doxorubicin, PHSM formulation resulted in 4-fold and 10-fold increase in doxorubicin level in the tumor over PHIM and the free drug, respectively. In mice bearing multidrug-resistant ovarian A2780/DOX^R^ s.c. xenografts, doxorubicin-loaded PHSM almost completely arrested tumor growth, whereas only modest inhibition was observed with the PHIM formulation. These studies strongly suggest that the combined mechanism of folate targeting and pH sensitivity of the mixed micelles contribute to enhanced drug delivery and efficacy in the tumor.

Tsai *et al.* reported a mixed micelle construct composed of folate-PEG-PLA and poly(2-HEMA-*co*-histidine)-*g*-PLA as a pH-sensitive carrier for doxorubicin [26]. Results from the NIR imaging study indicated that folate-coupled mixed micelles accumulated in the tumor to a higher extent, even though they were more rapidly eliminated from the circulation than the nontargeted micelles. In cervical adenocarcinoma HeLa s.c. xenograft-bearing mice, doxorubicin-loaded folate micelles displayed more potent tumor growth inhibition than free doxorubicin and the nontargeted micelles.

Zhang *et al.* evaluated folate-conjugated paclitaxel-loaded Pluronic P123/F127 mixed micelles in mice bearing s.c. MDR KB_v_ xenografts [27]. They confirmed an earlier finding that Pluronic P123/F127 mixed micelles prolonged the circulation time of paclitaxel compared to Taxol^®^, and revealed that functionalization of the micelles with folate slightly accelerate the clearance of the micellar drug. Notably, paclitaxel carried by folate-conjugated mixed micelles exerted strongest suppression in tumor growth among all studied paclitaxel formulations, which could be attributed to a combined effect of active targeting and the chemosensitization of MDR tumors by Pluronic copolymers. 

Syu *et al.* demonstrated the use of folate-functionalized poly(2-ethyl-2-oxazoline)-*b*-poly(d,l-lactide) (PEOz-PLA) micelles for the delivery of meta-tetra(hydroxyphenyl)chlorin (m-THPC), a clinically approved photosensitizer [28]. In KB s.c. xenograft-bearing mice, whereas photodynamic therapy caused similar tumor growth inhibition in mice receiving free m-THPC or the nontargeted m-THPC micelles, significantly more potent anticancer effect was observed in mice receiving folate-conjugated m-THPC micelles. Accordingly, m-THPC accumulation in the tumor tissues of mice treated with folate-modified micelles was nearly twice that of the free drug or the nontargeted micelles. 

### 3.2. Transferrin

Transferrin is a plasma protein that mediates the transport of iron into the cells by binding to the transferrin receptor (TfR) on the cell membrane. As a targeting moiety, transferrin triggers receptor-mediated endocytosis in cells that highly express TfR, which include many cancer types as well as the blood-brain barrier [39]. To improve the delivery of paclitaxel into the brain, Zhang *et al.* devised transferrin-conjugated micelles composed of PCL-*b*-poly(ethyl ethylene phosphate) (PCL-PEEP) [29]. They found that the concentration of paclitaxel in the brain tissue was almost doubled when the drug-loaded micelles was functionalized with transferrin, whereas the biodistribution of paclitaxel in other tissues were essentially unaltered. As a result, the anti-glioma efficacy of paclitaxel-loaded transferrin-conjugated micelles, as reflected by the prolonged survival of mice bearing intracranial glioblastoma U87MG xenografts, was significantly superior to those of the unmodified micelles and Taxol^®^.

### 3.3. Tumor-Targeting Peptides

Another intensely studied class of tumor-targeting ligands is RGD-containing peptides, which recognize integrins, a family of receptor proteins that are overexpressed in angiogenic tumor endothelial cells, as well as a wide variety of solid tumors [40]. Zhan *et al.* evaluated cyclic Arg-Gly-Asp-d-Tyr-Lys (cRGD) conjugated PEG-PLA micelles as drug carriers for paclitaxel. It was shown that while the drug-loaded PEG-PLA micelles and Taxol^®^ arrested tumor growth in mice bearing U87MG s.c. xenografts, cRGD-conjugated paclitaxel micelles exhibited the most potent tumor growth inhibition [30]. Notably, treatment with cRGD-conjugated paclitaxel micelles resulted in the longest survival time in intracranial U87MG tumor-bearing mice. 

Peptides containing Asn-Gly-Arg (NGR) motif have been shown to recognize aminopeptidase N, which is expressed in pericytes associated with the brain endothelial cells [41]. The cyclic NGR peptide CGCNGRC was conjugated to PEG-DSPE micelles to achieve targeted delivery of paclitaxel in a C6 glioma-bearing rat model [31]. Compared with the nontargeted paclitaxel-loaded PEG-DSPE micelles, NGR-modified PEG-DSPE micelles caused more potent tumor growth retardation, while free paclitaxel was ineffective.

l-SP5 (_L_(SVSVGMKPSPRP)) is a peptide that recognizes the tumor vasculature but not normal blood vessles [42]. As the retro-inverso form of L-SP5 using d-amino acids, d-SP5 (_D_(PRPSPKMGVSVS)) was found to be substantially more stable in serum than l-SP5 [32]. Interestingly, d-SP5 could not only bind to human endothelial HUVEC cells with higher affinity than l-SP5, it could also bind avidly to various tumor cells. In KB s.c. xenograft-bearing mice, doxorubicin-loaded d-SP5-PEG-DSPE micelles demonstrated over 3-fold increase in the tumor accumulation in comparison with doxorubicin carried by the nontargeted or l-SP5-linked micelles. The drug-loaded d-SP5-PEG-DSPE micelles, but not the nontargeted or l-SP5-conjugated PEG-DSPE micelles, pronouncedly improved the anticancer efficacy of doxorubicin.

OA02 peptide, which has a high binding affinity to α_3_ integrin on ovarian cancer cells, was utilized as a targeting ligand for paclitaxel-loaded PEG^5K^-CA_8_ micelles. Lam and his coworkers reported that the functionalization of PEG^5K^-CA_8_ micelles with OA02 notably enhanced the accumulation of micelles in the tumor [33]. In SKOV-3 s.c. tumor-bearing mice, paclitaxel-loaded OA02-PEG^5K^-CA_8_ micelles demonstrated the most potent antitumor efficacy with respect to the tumor growth inhibition and prolonged survival, when compared with Taxol^®^ and the nontargeted drug-loaded micelles. 

Cell penetrating peptides comprise short and usually basic amino acids-rich peptides that are able to rapidly internalized across cell membranes [43]. As a cell-penetrating peptide, TATp was conjugated to PEG-DSPE micelles and evaluated in 4T1 s.c. xenograft-bearing mice [34]. When injected intratumorly, paclitaxel-loaded TATp-PEG-DSPE micelles elicited more apoptotic cell death in the tumor than free paclitaxel or the plain drug-loaded micelles.

Lanreotide is an octapeptide analog of endogenous somatostatin with a high affinity for somatostatin receptor 2 (SSTR2), which is overexpressed in many tumor types [44]. Zheng *et al.* investigated lanreotide-conjugated PEG-PCL micelles as drug carriers for paclitaxel [35]. Two cancer cell lines, human lung cancer H446 (with high SSTR2 expression) and human breast cancer MCF-7(with low SSTR2 expression), were used in the study. They found that without active targeting, paclitaxel-loaded PEG-PCL micelles were less effective than Taxol^®^ in suppressing tumor growth in mice bearing either H446 or MCF-7 s.c. xenografts. In contrast, lanreotide-conjugated paclitaxelmicelles exhibited the strongest antitumor efficacy among all studied paclitaxel formulations in both tumor models. Interestingly, the active targeting micelles were more effective in H446 tumor than MCF-7 tumor, consistent with the notion of SSTR2-mediated drug uptake and action. Preferential accumulation of lanreotide-conjugated micelles in H446 tumor was evident from the biodistribution, NIR imaging and confocal microscopy studies.

LyP-1 (Cys-Gly-Asn-Lys-Arg-Thr-Arg-Gly-Cys) is a cyclic peptide that specifically binds to p32/gC1qR on the tumor cells and tumor lymphatic endothelial cells [45]. Wang *et al.* reported the delivery of artemisinin, a poorly water-soluble antimalarial drug with recently discovered anticancer activity, in LyP-1-modified PEG-PCL micelles [36]. NIR imaging study showed LyP-1-conjugated micelles could achieve enhanced and targeted accumulation in the tumor. Interestingly, LyP-1-conjugated micelles were found to home to the tumor lymphatic vessels, whereas the plain PEG-PCL micelles colocalized with the blood vessels. In an orthotopic breast cancer mouse model with highly metastatic MDA-MB-435S tumors, artemisinin-loaded LyP-1-conjugated micelles exhibited the most potent tumor growth inhibition compared with the free drug and the plain micelles.

AP peptide (CRKRLDRN) binds to IL-4 receptors, which are highly expressed in the breast tumor cells [46]. In a study by Wu *et al.* AP-conjugated pH-sensitive micelles composed of AP-PEG-PLA and MPEG-PAE were evaluated in mice bearing MDA-MB-231 s.c. xenografts [37]. Results from NIR imaging study showed that AP-modified micelles rapidly accumulated in the tumor and got retained for at least 24 h, whereas the unmodified micelles permeated into the tumor at a much slower rate, indicating active targeting via AP peptide increases the tumor targeting efficiency and retention of these micelles. Furthermore, doxorubicin-loaded AP-conjugated micelles showed superior tumor growth suppression over the untargeted micelles, both of which were markedly more efficacious than free doxorubicin. 

## 4. Cross-Linked Micelles Improve the Stability and Drug Delivery Efficiency

To minimize the premature release of drugs from their carriers during circulation in the blood stream, various strategies, such as photo-crosslinking, free radical polymerization and amide bond formation, have been devised to cross-link the shell or core of the polymeric micelles to prevent the self-assembled structures from dissociation [47]. One approach proven to be suitable for *in vivo* applications is to generate disulfide cross-linkage between thiolated block polymers, as exemplified by the studies listed in Table 3. These redox-sensitive cross-links are disrupted intracellularly by glutathione (GSH), an endogenous reducing agent of disulfide bond that has substantially higher concentration (1–10 mM) in cytoplasm than in extracellular fluid (1–10 μM), triggering the rapid destabilization of the cross-linked micelles and achieve efficient intracellular drug release [48]. 

Lee *et al.* reported recently that the stability of PEG-PLA micelles could be substantially enhanced by inserting a (Cys)_4_ peptide between PEG and PLA blocks, which allowed for disulfide cross-linking of the block polymers at the interface between the hydrophobic core and hydrophilic shell [49]. Results indicated that the DiO and DiI-loaded cross-linked PEG-(Cys)_4_-PLA micelles remained intact in the blood stream for at least 12 h, revealed by the intense FRET signals in the circulation. To further demonstrate the drug delivery capacity of these micelles, the pharmacokinetics of doxorubicin-loaded cross-linked PEG-(Cys)_4_-PLA micelles was studied in mice bearing lung cancer M109 s.c. xenografts. It was found that the cross-linked micelles drastically prolonged the circulation time of doxorubicin, which increased the drug accumulation in the tumor by 7-fold and decreased the drug uptake in the heart by 1.9 fold, compared to the non-crosslinked counterparts. As a result, doxorubicin carried by cross-linked PEG-(Cys)_4_-PLA almost completely suppressed the tumor growth at 2 mg/kg, a dose that was otherwise entirely ineffective for free doxorubicin and the non-crosslinked formulation. 

Lam and his coworker improved the stability of PEG^5k^-CA_8_ micelles by coupling cysteines onto the free amines of lysine residues (PEG^5k^-Cys_4_-L_8_-CA_8_). The introduction of cysteines into the telodendrimers allowed for the formation of disulfide cross-linked PEG^5k^-Cys_4_-L_8_-CA_8 _micelles (DCMs) [50]. They found that the DCMs had a longer circulation time and accumulate in SKOV-3 tumors to a higher level when compared to the non-crosslinked micelles (NCMs). As a result, the DCM formulation for paclitaxel was significantly more efficacious than either the free drug or the NCM formulation. The anti-tumor efficacy of paclitaxel-loaded cross-linked micelles was further enhanced by triggering the drug release in the tumor via the administration of N-acetylcysteine, a reducing agent that cleaves intramicellar disulfide bridges.

**Table 3:** Cross-linked micelles improve the stability and drug delivery efficiency.

**Polymer**	**Drug**	**Size (nm)**	**Tumor Model**	**Effect** *(compared to the free drug and/or non-crosslinked micelles)*	**Ref.**
PEG-(Cys)_4_-PLA	doxorubicin	–	s.c. M109	↑ drug levels in the tumor ↓ tumor growth	[49]
PEG^5K^-Cys_4_-L_8_-CA_8_	paclitaxel	28	s.c. SKOV-3	↓ tumor growth ↑ survival time	[50]
PEG-PLys-PPhe	docetaxel	59	s.c. MDA-MB-231	↑ drug levels in the tumor and normal organs ↓ tumor growth ↑ survival time	[51]
mPEG-*b*-p(HPMA_m_-Lac_n_)	doxorubicin	80	s.c. B16F10	↑ survival time	[52]

In a study by Koo *et al.* disulfide cross-linked PEG-PLys-PPhe micelles were evaluated as a novel delivery system for docetaxel [51]. The primary amines of PLys reacted with dithiobis(sulfosuccinimidylpropionate) (DTSSP) to form intramicellar disulfide cross-links. It was found that the cross-linked PEG-PLys-PPhe micelles displayed much higher stability and prolonged circulation time than the non-crosslinked micelles. In mice bearing MDA-MB-231 s.c. xenografts, the cross-linked micelles accumulated in the tumor and healthy organs to a greater extent than the unmodified micelles. Consequently, docetaxel-loaded cross-linked PEG-PLys-PPhe micelles demonstrated enhanced antitumor efficacy in tumor-bearing mice compared to free docetaxel and the non-crosslinked counterparts. 

Different from disulfide cross-linkage, Talelli *et al.* investigated core-crosslinked micelles composed of PEG-*b*-poly[*N*-(2-hydroxypropyl) methacrylamide-lactate] (mPEG-*b*-p(HPMA_m_-Lac_n_)) with doxorubicin methacrylamide (DOX-MA) covalently loaded into the micellar core [52]. Owing to the pH-sensitive hydrozone linker of DOX-MA, the release of doxorubicin from the cosslinked micelles was accelerated in acidic condition. Doxorubicin-loaded micelles significantly prolonged the survival of mice bearing B16F10 s.c. xenografts, in comparison with the mice treated with free doxorubicin.

## 5. Tumor-Specific Delivery of Plasmid DNA (pDNA) and Small Interference RNA (siRNA) Is Achievable via Polyplex Micelles

In addition to hydrophobic small-molecule anticancer drugs, polymeric micelles have also been designed to deliver nucleic acids *in vivo*, including both pDNA and siRNA [53,54,55]. Gene-coding pDNA are circular double-stranded DNA typically with 5000 base pairs. siRNA are double-stranded RNA consisting of ~21 base pairs, which potently abrogate the expression of target genes in a sequence-specific manner via inherent RNA interference processing [56]. Despite differences in molecular weight, charge ratio, stability and mechanism of action, all nucleic acid-based therapeutics must gain access to the cytoplasm (and further import into the nucleus for pDNA) in order to be effective. There are formidable challenges common to the delivery of pDNA and siRNA. First, naked pDNA and siRNA are highly unstable in the circulation and are rapidly eliminated from the body. Second, due to their large size and anionic nature, plasmid DNA and siRNA cannot diffuse across the cell membrane and require active internalization into cells by endocytosis. By utilizing cationic micelle-forming block polymers, negatively charged pDNA and siRNA under physiological pH can be entrapped within the micellar core via electrostatic interaction and form stable complexes (polyplex micelles). A number of micellar carriers have been devised and investigated with promising antitumor effect *in vivo* (Table 4). 

**Table 4:** Tumor-specific delivery of pDNA and siRNA is achievable via polyplex micelles.

pDNA or siRNA	Polymer	Size (nm)	Targeting Ligand	Tumor Model	Effects	Ref.
pDNA encoding sFlt-1	thiolated PEG-PLL (disulfide cross-linked)	116	–	s.c. BxPC-3	*Compared to the non-cross-linked micelles*, ↑ sFlt-1 expression in the tumor ↓ tumor growth ↓ vascular density	[57]
pDNA encoding sFlt-1	thiolated PEG-PLL (disulfide cross-linked)	104	cRGD	s.c. BxPC-3	*Compared to the non-targeted micelles,* ↓ pDNA level in blood ↑ pDNA level in the tumor ↓ tumor growth ↓ vascular density	[58]
pDNA encoding RGD4C-hTNF-α	PEG-SS-p[Asp(DET)]	80–90	–	i.p. SUIT-2	↑ hTNF-α expression in the tumor ↓ i.p. tumor burden ↑ survival time	[59]
VEGF/VEGFR2 siRNA	PEG-PLL(2IT)	45	cRGD	s.c. HeLa	↑ siRNA levels in blood and the tumor ↓ tumor growth	[60]
VEGF siRNA	siRNA-conjugated PEG & PEI	99	–	s.c. PC-3	↑ VEGF siRNA levels in blood and the tumor ↓ VEGF mRNA and protein levels in the tumor ↓ microvessel density ↓ tumor growth	[61]
AC siRNA	PEG-PCL-PPEEA	60	–	s.c. BT474	↓ AC mRNA and protein levels in the tumor ↓ tumor growth	[62]
Plk1 siRNA with paclitaxel	PEG-PCL-PPEEA	50	–	s.c. MDA-MB-435	co-localization of Plk1 siRNA and paclitaxel in the same tumor cells ↓ Plk1 protein in the tumor ↓ tumor growth	[63]

### 5.1. pDNA Delivery

Kataoka and his coworkers demonstrated that disulfide cross-linked polyplex micelles composed of thiolated PEG-*b*-poly(l-lysine) (PEG-PLL) block polymer could deliver pDNA encoding the soluble form of vascular endothelial growth factor (VEGF) receptor-1 (sFlt-1) *in vivo* [57]. *N*-succinimidyl 3-(2-pyridyldition)propionate (SPDP) was used to thiolate the ε-amino groups of the lysine residue, resulting in the decreased charge density. In mice bearing pancreatic cancer BxPC-3 s.c. xenografts, sFlt-1 pDNA-loaded polyplex micelles with 11% thiolation (B-SH11%) notably suppressed the tumor growth, which was associated with significantly elevated sFlt-1 levels in the tumor. In contrast, the polyplex micelles with either higher or lower thiolation/cross-linking (B-SH0%, B-SH5%, B-SH20% and B-SH36%) did not show any anticancer effect. These results strongly suggest that the balance between the density of cationic charge and the extent of disulfide cross-linking in PEG-PLL micelles is crucial for efficient pDNA delivery. In a follow-up study, the authors functionalized the disulfide cross-linked PEG-PLL polyplex micelles (B-SH15%) with cRGD ligand [58]. They found that, despite reduced circulation time, pDNA carried by cRGD-conjugated B-SH15% micelles achieved significantly higher accumulation in the tumor than the nontargeted micelles. As a result, augmented anticancer efficacy was observed in BxPC-3 s.c. xenograft-bearing mice.

In a study by Kumagai *et al.* polyplex micelles composed of PEG-disulfide-poly{*N*-[*N*-(2-aminoethyl)-2-aminoethyl]aspartamide} (PEG-SS-P[Asp(DET)]) were evaluated as a carrier system for pDNA encoding RGD4C-fused human tumor necrosis factor α (RGD4ChTNF-α) gene [59]. Disulfide linkage between PEG and P[Asp(DET)] allowed PEG to be detached upon intracellular reduction. In mice inoculated intraperitoneally with pancreatic carcinoma SUIT-2 cells, i.p. injection of PEG-SS-P[Asp(DET)] polyplex micelles resulted in about 5-fold higher hTNF-α expression than PEG-P[Asp(DET) in the tumor. Remarkably, a single dose of PEG-SS-P[Asp(DET)] polyplex micelles resulted in significantly reduced intraperitoneal tumor burden and prolonged survival, compared to saline or the unmodified PEG-P[Asp(DET)]-treated mice. 

### 5.2. siRNA Delivery

Interestingly, Kataoka *et al.* described another PEG-PLL-based polyplex micelle system for VEGF and VEGF receptor 2 (VEGFR2) siRNA, in which amines of PLL were modified with 2-aminothiolane (2IT) and the cRGD peptide was installed on the PEG terminus [60]. In contrast to SDDP, which introduced the sulfhydryl group by neutralizing cationic amines, the charge density of the PLL segment remained unchanged following thiolation by 2IT. The introduction of 2IT to the lysine side chains not only enhanced the binding affinity for siRNA, it also allowed for disulfide cross-links in the micelle core, which further imparted the stability in the micelle structure. Without increasing in nonspecific distribution of siRNA to normal tissues, the cRGD-linked siRNA micelles increased the accumulation of VEGFR2 siRNA in the tumor by 2-fold when compared to the naked siRNA and the nontargeted micelles, leading to the most potent anticancer efficacy in mice bearing cervical cancer HeLa s.c. xenografts.

Park and his coworkers reported that by conjugating VEGF siRNA with PEG, they were able to prepare stable siRNA-PEG/PEI polyplex micelles (polyelectrolyte complex) and achieve efficient siRNA delivery [61]. The siRNA-PEG conjugate via a disulfide link was uniquely designed to release the intact siRNA in reductive intracellular environment. In prostate cancer PC-3 xenografts-bearing mice, VEGF siRNA-PEG/PEI polyelectrolyte complex markedly downregulated VEGF gene expression at both the transcriptional and protein levels in the tumor, leading to reduced tumor microvessel density and the most potent tumor growth suppression compared to the naked siRNA and siRNA/PEI complex. The enhanced gene silencing by siRNA-PEG/PEI micelles was well correlated with the prolonged circulation time of siRNA and increased accumulation in the tumor via the EPR effect. 

In a study by Wang *et al.* a polyplex micelle (micelleplex) system comprised of a triblock copolymer PEG-*b*-PCL-*b*-poly(2-aminoethyl ethylene phosphate) (PEG-PCL-PPEEA) was evaluated for siRNA delivery [62]. Whereas the hydrophobic interaction between PCL blocks induced the micellar core formation, the positively charged PPEEA segment served as the siRNA binding site. It was shown in a breast cancer BT474 s.c. xenograft murine model that acid ceramidase (AC) siRNA-loaded micelleplex markedly downregulated AC gene expression in the tumor cells and retarded the tumor growth. Moreover, the same PEG-PCL-PPEEA micelle construct was utilized to concurrently deliver polo-like kinase 1 (Plk1) siRNA and paclitaxel [63]. In mice bearing MDA-MB-435 s.c. xenografts, the micelleplex carrying both Plk1 siRNA and paclitaxel was shown to simultaneous deliver the two agents at the designated ratio to the same tumor cells and synergistically suppress tumor growth, which was significantly more potent than Taxol^®^. Intriguingly, co-administration of micelles separately loaded with siRNA and paclitaxel showed much less inhibition of tumor growth with no synergistic effect. This study underscores the potential of polymeric micelles for combined drug and siRNA therapy. 

## 6. Summary

The superior flexibility in polymer design coupled with the general simplicity of physical drug entrapment has distinguished polymeric micelles as versatile and effective drug carriers for cancer therapy. Herein, we have briefly summarized the recent progresses (mostly within the past 5 years) that have explored the use of polymeric micelles in preclinical tumor models. The promising results gained from these studies highlight the increasingly sophisticated designs of micelle constructs to overcome the biological barriers and achieve targeted delivery of anticancer drugs to the tumor, which have provided crucial stepping stones to further develop micellar nanocarriers for targeted cancer therapy.
